# Should Incretin Agonist-Based Drugs be Considered for First Line Antihypertensive Therapy?

**DOI:** 10.1007/s11906-026-01374-7

**Published:** 2026-05-07

**Authors:** Dominik Kylies, Leonie Dreher, Ulrich O. Wenzel

**Affiliations:** 1 III. Department of Medicine, University Medical Center Hamburg-Eppendorf, Hamburg, Germany; 2 Hamburg Center for Kidney Health (HCKH), University Medical Center Hamburg- Eppendorf, Hamburg, Germany

**Keywords:** Incretin-based therapies, Blood pressure reduction, Hypertension management, Weight loss–independent mechanisms

## Abstract

**Purpose of Review:**

This review evaluates the antihypertensive potential of next-generation incretin-based therapies, including GLP-1 receptor agonists and dual GLP-1/GIP receptor agonists. It examines their effects on blood pressure reduction, underlying mechanisms, clinical benefits, and implications for future guidelines.

**Recent Findings:**

Recent large-scale trials demonstrate that incretin-based therapies such as semaglutide and tirzepatide significantly reduce body weight, blood pressure, and cardiovascular outcomes. Mediation analyses indicate that weight loss explains a substantial proportion of blood pressure reduction, while direct effects on vascular function, renal sodium handling, and neurohumoral pathways also contribute. These effects are consistent across diverse populations, including individuals without overt hypertension.

**Summary:**

Incretin-based therapies represent a promising approach in hypertension management, combining metabolic and cardiovascular benefits. Despite the lack of trials with blood pressure as a primary endpoint, current evidence supports their use in selected high-risk populations and suggests an emerging role in future guideline recommendations.

## Introduction

Over the last decades, advancements in antihypertensive treatments have been modest. In the mid-1990s, angiotensin II type 1 receptor (AT1)-blockers have been introduced followed by the introduction of the largely unsuccessful renin inhibitors in the 2000s. In recent years, various endothelin receptor antagonists have been evaluated as antihypertensive agents and very recently siRNA-based angiotensinogen-targeting, atrial natriuretic peptide analogues and novel aldosterone synthase inhibitors have been introduced [1]. Invasive procedures such as renal denervation represent another strategy for antihypertensive treatment. Some studies demonstrated that renal denervation can lead to a modest additional reduction in blood pressure in patients with resistant hypertension as well as in treatment-naïve patients. While studies on novel antihypertensive agents and renal denervation demonstrated significant blood pressure reductions, none were sufficiently powered to show a decrease in cardiovascular endpoints. Ideally, for future blood pressure treatment, the treatment should not only lower blood pressure but also significantly modify cardiovascular risk, morbidity and mortality.

One relatively novel medication class that checks these boxes are next-generation incretin-based therapeutics like GLP-1 RAs (glucagon-like peptide-1 receptor agonists, for example semaglutide) and dual GLP-1/GIP (glucose-dependent insulinotropic polypeptide, for example tirzepatide). Originally developed and widely used as antidiabetic agents, and later adopted for weight loss [2], these therapies remain underutilized in the management of hypertension. Fittingly, a current NEJM review article focusing on GLP-1 RAs hardly mentions blood pressure reduction [3]. Despite still being under recognized as potentially meaningful antihypertensive medications, there is growing interest in the hypertension community [4]. However, several key questions remain: Are the observed blood pressure–lowering effects solely attributable to weight loss, or are they mediated by direct, tissue-specific mechanisms independent of weight reduction [5]? Furthermore, how will these findings be incorporated into future clinical guidelines? In this review, we summarize the current evidence on underlying pathophysiological mechanisms, tissue-specific effects, and clinical blood pressure–lowering efficacy, as well as their implications for guideline development.

## Mechanisms of Blood Pressure Reduction

One key question regarding the mechanisms of blood pressure reduction in next-generation incretin-based therapeutics is to which extent their effects are mediated via weight loss versus tissue-derived direct effects. Another important question relates to the mechanisms of reductions in major adverse cardiovascular events and organ-protection of these drugs. While it has been proposed that a substantial proportion of the end organ protection may be driven by weight reduction and direct tissue effects of these drugs, their exact mechanisms in many cases are still incompletely understood [5, 6].

**Fig. 1:**
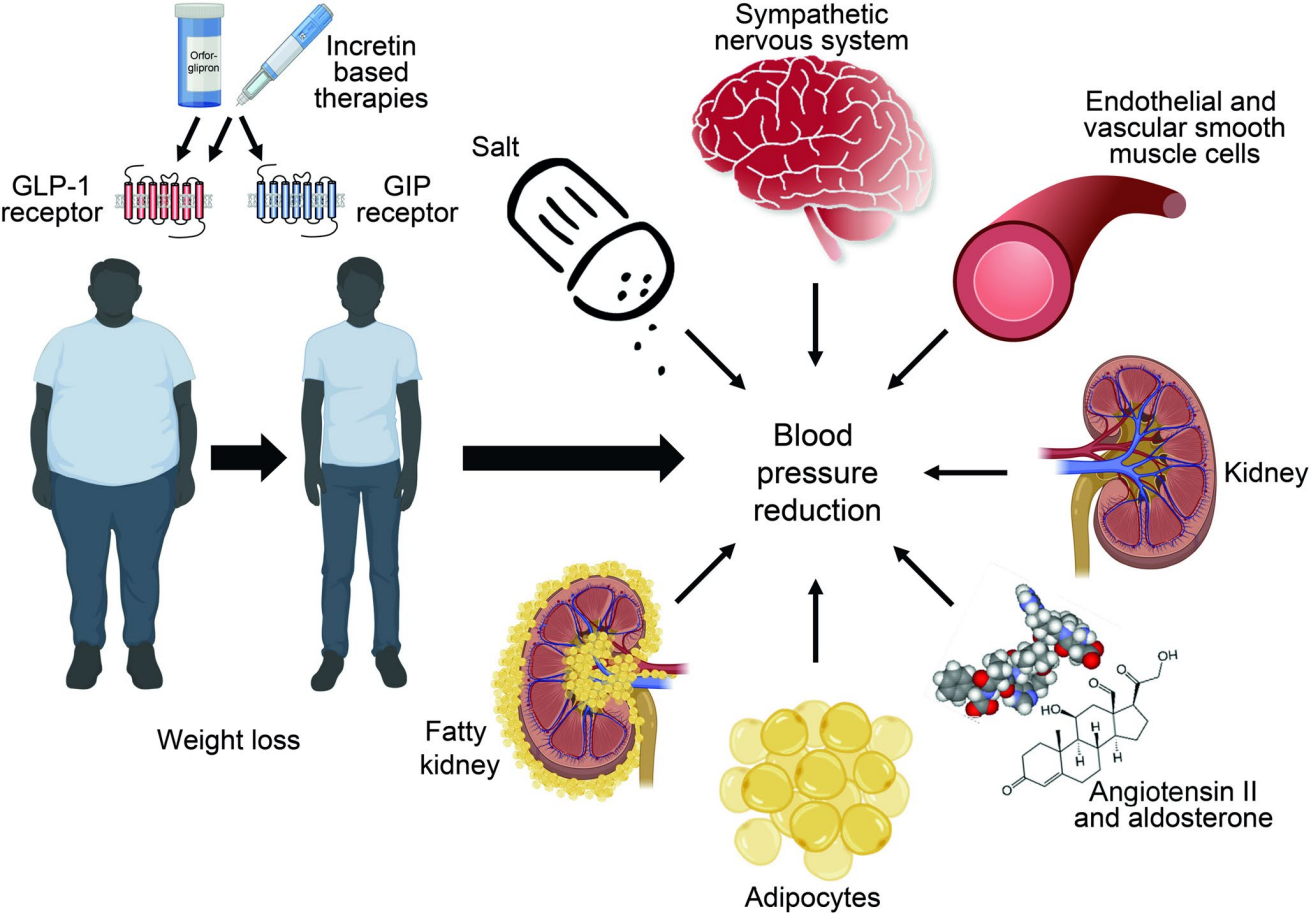
Mechanisms of blood pressure reduction by incretin-based therapies

Weight loss achieved through next-generation incretin-based therapies is mediated by three primary mechanisms: increased satiety, and a reduction in both gastric emptying and hunger. However, mechanisms beyond weight reduction likely also contribute to blood pressure lowering. This may be explained by the widespread expression of the GLP-1 receptor across cardiovascular tissues. Consistently, GLP-1 receptor agonists have demonstrated sustained antihypertensive effects in several non-diabetic and non-obese preclinical models of hypertension [7]. More recently, several pathways involved in the pathogenesis of hypertension have been identified that align with the mosaic theory of arterial hypertension [8–10]. GLP-1 receptor agonists appear to modulate nearly all of these components; the mechanisms underlying their blood pressure-lowering effects are illustrated in Fig. 1. Incretin-based therapies reduce overall food consumption, which consequently results in a lower intake of dietary salt. The sympathetic nervous system is a key contributor to the pathogenesis of hypertension, and GLP-1 signaling may play an important modulatory role in this pathway [7]. The GLP-1 receptor is widely expressed within the central nervous system, particularly in the hypothalamus and brainstem which are key regions involved in autonomic and blood pressure regulation [11]. Emerging evidence from experimental models suggests that sustained activation of brainstem GLP-1 receptors plays important roles in directly lowering blood pressure and reducing sympathoexcitation [12]. Importantly, GLP-1 receptor agonists can cross the blood–brain barrier, and GLP-1 is also synthesized within the brain itself [13].

Endothelial and vascular smooth muscle cells also express the GLP-1 receptor and experimental studies showed that pharmacological GLP-1 receptor activation may attenuate hypertension-related injury by reducing vascular inflammation via selective endothelial mechanisms [14]. GLP-1 signaling contributes to endothelial nitric oxide production and bioavailability, thereby supporting the maintenance of vascular tone. In addition, it modulates inflammatory cell infiltration and reduces the generation of reactive oxygen species [15]. Notably, GLP-1 may also attenuate vascular remodeling, a key feature of hypertension [16]. Renal sodium handling, specifically reabsorption and excretion, is central to extracellular fluid balance and blood pressure regulation. GLP-1 receptor agonists have been shown to enhance natriuresis. These diuretic and natriuretic effects are accompanied by reduced urinary proton excretion, indicating involvement of the Na⁺/H⁺ exchanger NHE3 [17]. Consistently, in patients with diabetic nephropathy, treatment with semaglutide has been associated with reductions in albuminuria, a slower decline in eGFR, and improved overall cardiovascular outcomes [18].

Another mechanism by which the GLP-1 axis may promote antihypertensive effects is angiotensin. GLP-1 has been shown to inhibit components of the renin–angiotensin system, with evidence supporting crosstalk between GLP-1 and angiotensin II signaling pathways [7].

Finally, incretin-based therapies reduce adipose tissue mass by decreasing both adipocyte size and number. Aldosterone production by adipocytes has been previously described [19, 20]. Furthermore, leptin secreted by adipocytes has been shown to enhance aldosterone production in the adrenal glands of female mice by directly stimulating aldosterone synthase expression [21, 22].

As recently highlighted in an editorial by Hall et al. [23], increased perirenal and renal hilar fat have been associated with chronic kidney disease and hypertension [24, 25]. Proposed mechanisms include mechanical compression of renal vessels and nerves, induction of glomerular hyperfiltration, activation of the renin–angiotensin–aldosterone system, and promotion of inflammation through local cytokine release [26]. The effects and clinical implications of GLP-1 receptor agonists on kidney-specific fat are currently being investigated in several studies (NCT04865770, NCT05536804, NCT05936151) [27–29].

But to which extent are these mechanisms responsible for antihypertensive effects? Recent mediation analysis suggested that the associated weight loss appears to play the most important but not only role in the observed blood pressure reductions. Mediation analyses are statistical methods used to determine whether an independent variable (in this case, treatment with a next-generation incretin-based therapeutic) affects a dependent variable (i.e., blood pressure) directly, or whether, and to what extent, this effect is mediated by a third variable (i.e., weight loss). For both tirzepatide and semaglutide, recent mediation analyses were performed. For tirzepatide, in patients with obesity it was concluded that weight loss explained 68% of the reduction in systolic BP [30]. Similarly, for semaglutide, 89% of the reduction in systolic blood pressure was explained by weight loss [31].

Another hint can be derived from a head-to-head trial comparing the weight reduction efficacy of semaglutide and tirzepatide in patients with obesity. The change in body weight was − 20.2% with tirzepatide compared to -13.7% with semaglutide. Accordingly, the reduction in systolic blood pressure was − 10.2 mmHg in the tirzepatide group versus − 7.7 mmHg in the semaglutide group, suggesting that the blood pressure reduction follows the extent of weight loss [32]. A similar conclusion can be derived from the REDEFINE Trial [33]. CagriSema is a fixed-dose combination of semaglutide and cagrilintide that achieved a superior body weight reduction of -15.6% in the REDEFINE 1 trial, significantly outperforming semaglutide (-5.1%), cagrilintide (-8.1%), and placebo (-0.8%). Correspondingly, systolic blood pressure decreased by 10.9 mmHg with CagriSema versus 7.3 mmHg with semaglutide and 2.8 mmHg with placebo, with 39.6% of patients even reducing or stopping their antihypertensive medication. These results confirm that the antihypertensive effect once again closely follows the extent of weight loss [34].

## Clinical Evidence for Blood Pressure Reduction

Besides analyzing glycemic control, initial clinical trials of semaglutide and tirzepatide in patients with diabetes, as well as in individuals with obesity without diabetes, focused on weight reduction as the main endpoint. Subsequent studies expanded to cardiovascular outcome trials, addressing a broader range of comorbidities, including heart failure with reduced (HFrEF) and preserved ejection fraction (HFpEF) and chronic kidney disease. While blood pressure outcomes have been reported across most of these trials, they were not the primary focus. As none of these studies were designed with blood pressure reduction as a primary end point a substantial amount of bias must be considered when interpreting the reported blood pressure changes. Specifically, the number and type of antihypertensive medications were often not systematically documented or analyzed. This is important because these potential reductions in antihypertensive medications may lead to an underestimation of the blood pressure effects of these next-generation incretin-based therapeutics. Indeed, an individual patient–level meta-analysis from Kennedy et al. demonstrated that de-escalation of antihypertensive therapy occurred significantly more frequently in patients treated with semaglutide [31]. Notably, this effect was most pronounced in patients with resistant hypertension, where 27% of those treated with semaglutide were able to reduce their antihypertensive therapy compared with only 3% in the placebo group. Irrespective of the presence of baseline hypertension, they found an overall reduction of 5 mmHg.

Generally, in many of the available studies, baseline blood pressure was not elevated, either due to well-controlled participants or inclusion of normotensive individuals, depending on the trial design and population. This makes the observed reductions in blood pressure particularly remarkable. The changes in body weight, baseline systolic blood pressure and blood pressure reductions in the major placebo-controlled semaglutide and tirzepatide studies are shown in the Table 1 adopted from Dreher et al. [4].

**Table 1:** Placebo-corrected effects of incretin-based therapies on body weight and systolic blood pressure in majorrandomized clinical trials

Medication	Trial name	Inclusion	Dose (mg)	Baseline BMI	Placebo-corrected body weight change (%)	Baseline SBP (mmHg)	Placebo-corrected SBP change (mmHg)	Ref.
Semaglutide	SUSTAIN	Type 2 diabetes	1*	32.9	−4.78	136	−2.6	[35]
	FLOW	Type 2 diabetes with CKD	1	31.9	−4.54	139	−2.2	[18]
	STEP 1	BMI > 27 with comorbidity or BMI > 30	2.4	37.8	−12.5	126	−5.1	[36]
	STEP 3	BMI > 27 with comorbidity or BMI > 30	2.4	38.1	−10.6	124	−1.9	[37]
	SELECT	BMI > 27 with comorbidity	2.4	33.3	−8.51	131	−3.3	[38]
	STEP-HFpEF	HFpEF with BMI > 30	2.4	37.2	−10.7	133	−2.9	[39]
Oral Semaglutide	PIONEER 6	Type 2 diabetes with CVD, CKD or cardiovascular risk factors	14	32.3	−3.72	135	−2.6	[40]
Orforglipron	n/a	BMI > 27 with comorbidity or BMI > 30	45*	38.7	−12.4	127	−9.9	[41]
Tirzepatide	SURPASS-4	Type 2 diabetes with BMI > 25 with cardiovascular disease or high risk	15*	32.5	−15.1	134	−6.1	[42]
	SUMMIT	HFpEF with BMI > 30	15	38.3	−11.7	128	−4.7	[43]
	SURMOUNT-1	BMI > 27 with comorbidity or BMI > 30	15*	38.1	−17.8	123	−6.4	[44]
	SURMOUNT-1	Prediabetes and BMI > 27 with comorbidity or BMI > 30	15*	39.2	−18.4	125	−8.7	[45]
Semaglutide/ Cagrilintide	REDEFINE 1	BMI > 27 with comorbidity or BMI > 30	2.4/2.4**	37.9	−17.3	127	−6.7	[46]

*In studies applying multiple medication dosages, the results from the highest dosage is displayed. **Placebo-corrected body weight change and SBP change were displayed for the combined Semaglutide/ Cagrilintide-group vs. placebo

## Cardiovascular Endpoints of Novel Incretin Mimetics and Their Reflection in Future Guidelines

Angiotensin-converting enzyme inhibitors or angiotensin II receptor blockers, calcium channel blockers, and thiazide or thiazide-like diuretics currently are the first-line mainstay of antihypertensive treatment as recommended by guidelines from the American College of Cardiology/American Heart Association, European Society of Hypertension, and European Society of Cardiology. In addition, the European Society of Hypertension additionally includes β-blockers into their recommendations.

The foundation of these guideline recommendations are grounded in evidence from studies showing blood pressure reduction as a surrogate endpoint as well as reductions in cardiovascular outcomes in placebo-controlled trials. In line with our initial argument that over the last decades little progress has been made in the development of novel antihypertensive drugs, many of these studies are relatively dated, and their design and methodological rigor would, in many cases, not fully align with contemporary standards. On the other hand, next-generation incretin mimetics are supported by large-scale, high-quality trials involving substantial patient populations. While recent hypertension guidelines mention incretin-based therapies, their recommendations remain somewhat vague. This can be explained because most of the high-quality data and the subsequent clinical discussion emerged only after these guidelines were published.

This raises an important and somewhat provocative question as to whether GLP-1 and dual GLP-1/GIP receptor agonists may, in fact, represent some of the most comprehensively studied therapies with antihypertensive properties available.

From an antihypertensive perspective, based on the available and robust data from large clinical trials, their use in patients with hypertension and type 2 diabetes [35] as well as in those with hypertension and a BMI > 27 [38], is well justified, as they not only lower blood pressure but also improve hard cardiovascular outcomes.

Although direct comparisons are limited, the magnitude of blood pressure reduction observed with these agents is comparable to, or even exceeds, that achieved with many established antihypertensive drugs and interventions [47]. Recent mediation analyses suggest that 70–80% of the blood pressure–lowering effect is attributable to weight loss, raising the question of whether it is reasonable to use these therapies in lean patients with hypertension [31, 34]. However, these analyses do not address whether the improvement in cardiovascular outcomes is likewise mediated solely by weight reduction. It would therefore be of interest to develop new incretin‑based therapies that lower blood pressure and improve cardiovascular outcomes without inducing significant weight loss.

How will these medications affect future guidelines? One could argue that the data, at least for distinct patient cohorts, is sufficient to be classified as IA. This is because of the strong evidence and broad consensus for being effective, beneficial and clinically useful on the one side and supported by high quality data from multiple large, randomized placebo-controlled clinical trials on the other side. However, one could also argue against that by saying that currently no trial has evaluated blood pressure as a primary endpoint.

Notably, the title of the recent European Society of Cardiology guideline has shifted from “management of arterial hypertension” to “management of elevated blood pressure and hypertension.” This reflects the recognition that cardiovascular risk associated with blood pressure exists along a continuous spectrum rather than a strict dichotomy between normotension and hypertension. It also reflects a more holistic and multimodal approach to treating patients with elevated blood pressure. Emerging data further supports the benefit of blood pressure reduction even in individuals with high cardiovascular risk whose values fall below traditional diagnostic thresholds for hypertension [48]. In this context, it is particularly noteworthy that semaglutide appears to lower blood pressure to a similar extent in both normotensive and hypertensive individuals [31]. Overall it is likely that future iterations of these guidelines will incorporate next-generation incretin mimetics in one form or another.

## Sex Differences

Women have traditionally been underrepresented in hypertension trials, typically comprising only about 27% to 40% of participants in major studies. Although contemporary guidelines highlight the need to account for sex and gender differences, this gap persists—as illustrated by the relatively low proportion of women in the 2023 zilebesiran trial [49]. In contrast, trials evaluating incretin-based receptor agonists tend to enroll a higher percentage of female participants, suggesting that their findings are more readily generalizable to women.

## Limitations

The most striking limitation is that these trials were not designed with hypertension as the primary endpoint, thereby introducing a potential source of bias: Many trials of incretin-based therapies provide limited detail on concomitant medication adjustments, restricting the ability to assess how such changes may have influenced blood pressure outcomes. Moreover, most studies did not specifically enroll patients with hypertension or excluded those with more severe forms, highlighting the need for dedicated trials in individuals with obesity and difficult-to-treat or resistant hypertension to better define the role of incretin-based receptor agonists in this setting.

In addition, key factors such as changes in dietary intake and 24-hour urinary sodium excretion were often not evaluated, precluding assessment of the contribution of dietary factors, including salt intake, to observed blood pressure effects.

For many patients, incretin-based receptor agonists require long-term use, as their benefits tend to, at least partially, wane after discontinuation [50]. While this may be perceived as a limitation, it is important to note that the same principle applies to conventional antihypertensive therapies, where blood pressure typically rises once treatment is stopped.

Finally, there are barriers to consider. Incretin-based therapies are currently much more expensive than established, generic antihypertensive drugs. In addition, their safety profile is characterized by mild-to-moderate gastrointestinal adverse events.

## Conclusion

Modern antihypertensive agents should lower blood pressure and improve meaningful clinical outcomes alike. Their effect should be demonstrated repeatedly across large, placebo-controlled randomized trials designed for outcome evaluation, ensuring that trials are performed evenly across both sexes. They should be safe, at least metabolically neutral with respect to lipid and glucose parameters and, to improve patient compliance, ideally have a long half-life. Next-generation incretin-based therapeutics have the potential to check many of these boxes: They not only lower blood pressure but also have an overall favorable safety profile, reduce body weight, improve metabolic effects, particularly glycemic control, and may reduce cardiovascular events and all-cause mortality in multiple large randomized controlled trials in both patients with and without diabetes. However, it must be noted that they were not formally tested for blood pressure reduction as a primary outcome. Despite this, the emerging evidence is compelling: in patients with type 2 diabetes or a BMI > 27 accompanied by hypertension, GLP-1 receptor agonists rank among the most promising antihypertensive therapies currently available.

## Perspectives and Future Directions

Despite growing evidence supporting the antihypertensive effects of incretin-based therapies, several key questions remain. Most importantly, no randomized trial has yet evaluated blood pressure reduction as a primary endpoint. Consequently, dedicated hypertension trials are essential to precisely define their role within treatment algorithms, especially in non-obese populations and patients with treatment-resistant hypertension.

Beyond weight loss-dependent effects, further mechanistic studies are needed to uncover tissue-specific pathways, including vascular, renal, and neurohumoral mechanisms. Clinical research should also include ‘non-responders’ to weight loss and provide direct comparisons between incretin mimetics and lifestyle interventions or bariatric surgery at equivalent levels of weight loss. Ideally, these should be conducted as phase 3 trials designed to demonstrate a reduction in hard cardiovascular endpoints.

Furthermore, the impact of these therapies on antihypertensive medication burden and long-term adherence deserves investigation, particularly in individuals at high cardiovascular risk without overt diabetes. Taken together, these considerations suggest that incretin-based therapies may evolve from primarily metabolic agents to integral components of cardiovascular risk management, potentially reshaping future hypertension guidelines. 

Next-generation incretin-based therapeutics lower blood pressure through both weight loss–dependent and weight loss–independent mechanisms. Weight reduction contributes to blood pressure improvement via metabolic and hemodynamic changes. In addition, direct tissue effects involve multiple organ systems, including reduced intake of salt, modulation of the sympathetic nervous system, improvement of endothelial and vascular smooth muscle cell function, reduced tubular sodium reabsorption, and suppression of the renin–angiotensin–aldosterone system. Furthermore, reductions in adipose tissue mass and ectopic fat deposition, including perirenal fat (“fatty kidney”), may contribute to blood pressure lowering. These complementary pathways collectively result in improved blood pressure control. Some elements of this figure were created with BioRender.com.

Shown are selected placebo-controlled trials evaluating next-generation GLP-1 receptor agonists (semaglutide, oral semaglutide and orforglipron) and the dual GLP-1/GIP receptor agonist tirzepatide across various patient cohorts. Reported are baseline body mass index (BMI), baseline systolic blood pressure (SBP), and placebo-corrected changes in body weight and SBP. For trials investigating multiple dose levels, only results from the highest tested dose are presented. The table was adopted from Dreher et al. [4]. CKD = chronic kidney disease; CVD = cardiovascular disease; HFpEF = heart failure with preserved ejection fraction; SBP = systolic blood pressure.

## Data Availability

No datasets were generated or analysed during the current study.
