# Correction to: Should Incretin Agonist-Based Drugs be Considered for First Line Antihypertensive Therapy?

**DOI:** 10.1007/s11906-026-01377-4

**Published:** 2026-06-29

**Authors:** Dominik Kylies, Leonie Dreher, Ulrich O. Wenzel

**Affiliations:** 1 III. Department of Medicine, University Medical Center Hamburg-Eppendorf, Hamburg, Germany; 2 Hamburg Center for Kidney Health (HCKH), University Medical Center Hamburg- Eppendorf, Hamburg, Germany

**Correction to: Current Hypertension Reports (2026) 28:25**

10.1007/s11906-026-01374-7

The original online version of this article was revised. In this article the title was incorrectly given as ‘Should Incretin Agonist-Based Brugs be Considered for First Line Antihypertensive Therapy?’ but should have been ‘Should Incretin Agonist-Based Drugs be Considered for First Line Antihypertensive Therapy?‘.

The original article has been corrected.

